# LONELINESS AND DEPRESSION: EXAMINING THE MODERATING EFFECTS OF RESILIENCE RESOURCES

**DOI:** 10.1093/geroni/igac059.2614

**Published:** 2022-12-20

**Authors:** Xiaoyan Zhang, Merril Silverstein

**Affiliations:** Syracuse University, Syracuse, New York, United States; Syracuse University, Syracuse, New York, United States

## Abstract

Loneliness is highly prevalent among older adults and can negatively influence their mental health. However, less is known about the factors that might mitigate the effects of loneliness on mental health outcomes such as depression. We propose that resilience resources may serve as potential protective factors that buffer the impact of loneliness on depressive symptoms. This study aimed to (a) test the effects of loneliness, as well as resilience factors of perceived family support and optimism, on depressive symptoms, and (b) examine resilience factors as moderators of the relationship between loneliness and depressive symptoms. Participants derived from the Health and Retirement Study (HRS), a nationally representative sample of the population aged 50 years and older. The analytic sample was selected with baseline measurements in either 2006 or 2008 and two follow-ups across four-year intervals (N= 7,336). Structural equation modeling with latent variables and interaction terms was used to investigate study aims. Results revealed that (a) loneliness was significantly related to an increase in depressive symptoms; both perceived family support and optimism significantly reduced depressive symptoms, and (b) the link between loneliness and depressive symptoms was weaker when older adults reported higher levels of perceived family support and greater optimism. This study highlights the important protective roles played by perceived family support and optimism in reducing the adverse impact of loneliness on depression. Results suggest that strengthening family support and fostering optimism might be promising avenues for improving mental health in older adults particularly among those experiencing loneliness.

